# Highlight: How a Butterfly Tree Becomes a Web

**DOI:** 10.1093/gbe/evab143

**Published:** 2021-07-22

**Authors:** Casey McGrath

Evolution is often portrayed as a tree, with new species branching off from existing lineages, never again to meet. The truth however is often much messier. In the case of adaptive radiation, in which species diversify rapidly to fill different ecological niches, it can be difficult to resolve relationships, and the phylogeny (i.e., evolutionary tree) may look more like a web than a tree. This is because lineages may continue to interbreed as new species are established, and/or they may diverge and then rehybridize, resulting in genetically mixed populations (known as admixture). Even after species diverge, the introduction of genes from one species to another (known as introgression) can occur. All of this results in a network of related species, rather than a simple tree. The extent to which these processes occur and their evolutionary and genomic impacts are not well understood, partially due to the “tree-like” assumptions of the models that are used to construct phylogenies. In a new study in *Genome Biology and Evolution* titled “Rampant genome-wide admixture across the *Heliconius* radiation,” Krzysztof Kozak of the University of Cambridge and colleagues demonstrate the key role that interspecific gene flow played in the continent-wide adaptive radiation of the *Heliconius* butterflies (Kozak et al. 2021). This study adds to the rich literature on *Heliconius*, a genus that provided some of the earliest evidence for the theory of evolution thanks to their distinctive wing patterns and colors, which help warn predators of their toxic nature.

According to Kozak et al., “the Neotropical *Heliconius* butterflies present an excellent opportunity to study the incidence and importance of gene flow in a recent adaptive radiation, due to the natural propensity of *Heliconius* and the sister genus *Eueides* to produce hybrids in the wild.” In addition, the genes controlling their wing patterns are likely to be prime targets for selection and introgression, allowing different poisonous species to mimic each other and thus reinforcing the warning signal to predators. 

The study included genomic data from 145 individuals, representing 40 of the 47 recognized *Heliconius* species and 6 of the 12 *Eueides* species, allowing a comprehensive investigation into departures from a strict tree model. The analysis revealed several discrepancies in the evolutionary history of individual genes, suggesting the possibility of extensive gene flow among lineages. Overall, the authors uncovered 13 instances of interspecific gene flow across the phylogeny, revealing a pattern of gene sharing that includes all of the major clades of *Heliconius*. “We found that gene flow between species, previously documented in a few closely related species, has been common across the group for millions of years,” notes Dr Kozak, “including both existing and ancestral lineages.”

**Fig. 1.**
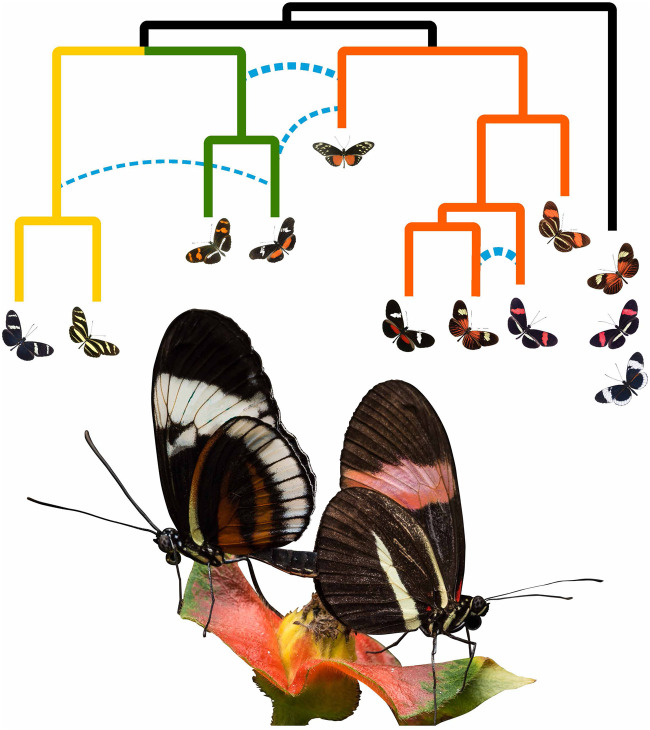
Above: A coalescent phylogenetic network of the *H. erato/clysonymus/sara* clade of *Heliconius* butterflies. Gene flow between species is indicated with dotted blue lines. Below: Butterflies of two different species (*Heliconius cydno chioneus* and *H. melpomene rosina*) mating on a *Psychotria poeppigiana* flower in Gamboa, Panama. Image by Krzysztof Kozak and Jorge Aleman. Photo credits: Luca Livraghi, Michel Cast.

Intriguingly, when analyzing genes known to be involved in wing pattern and color, the authors found evidence for complex patterns of gene flow across several lineages (fig. 1), supporting previous reports and also identifying new cases of introgression. According to Dr Kozak, “this provides further strong evidence that hybridization has been an important mechanism in the evolution of wing patterns, with sharing of relevant genes among many lineages allowing *Heliconius* to warn off avian predators.” This makes *Heliconius* one of only a few known examples of a lineage that has experienced adaptive introgression of multiple genes across several different species.

In the future, Dr Kozak et al. plan to use what they have learned about *Heliconius* to model their phylogenies as networks, rather than trees, allowing them to better understand the evolution of other butterfly traits, “from spatial learning to diverse arrays of pheromones and defensive toxins.” In addition, they hope to relate their results to the geographic distribution of *Heliconius*: “We need to explore geographic variation and study both the incidence of hybridization (individuals of different species mating) and the levels of gene flow (genomic signature of mixing) between various populations, which so far has been done only for a few species.” This type of work is likely to pose a considerable challenge, however, as many species and populations of *Heliconius* are rare and found only in remote locations, necessitating considerable field work.

As comprehensive genomic data sets continue to expand, Dr Kozak hopes to investigate other organisms to see how widespread such interspecific gene flow may be. “As always in evolutionary biology, we need to ask how much our conclusions apply to other taxa. Very few butterflies, or insects indeed, have so far been studied in this depth: it remains to be seen how much evidence for genome-wide admixture we can find throughout the extreme diversity of insects.”
